# *Trichoderma*: The “Secrets” of a Multitalented Biocontrol Agent

**DOI:** 10.3390/plants9060762

**Published:** 2020-06-18

**Authors:** Monika Sood, Dhriti Kapoor, Vipul Kumar, Mohamed S. Sheteiwy, Muthusamy Ramakrishnan, Marco Landi, Fabrizio Araniti, Anket Sharma

**Affiliations:** 1 School of Bioengineering and Biosciences, Lovely Professional University, Jalandhar-Delhi G.T. Road (NH-1), Phagwara, Punjab 144411, India; monika.11816033@lpu.in (M.S.); dhriti.21851@lpu.co.in (D.K.); 2 School of Agriculture, Lovely Professional University, Delhi-Jalandhar Highway, Phagwara, Punjab 144411, India; vipul.19845@lpu.co.in; 3 Department of Agronomy, Faculty of Agriculture, Mansoura University, Mansoura 35516, Egypt; salahco_2010@mans.edu.eg; 4 State Key Laboratory of Subtropical Silviculture, Zhejiang A&F University, Hangzhou 311300, China; ramky@zafu.edu.cn; 5 Department of Agriculture, University of Pisa, I-56124 Pisa, Italy; 6 CIRSEC, Centre for Climatic Change Impact, University of Pisa, Via del Borghetto 80, I-56124 Pisa, Italy; 7 Dipartimento AGRARIA, Università Mediterranea di Reggio Calabria, Località Feo di Vito, SNC I-89124 Reggio Calabria, Italy; fabrizio.araniti@unirc.it

**Keywords:** abiotic stress tolerance, antagonism, antibiosis, biocontrol, fungi, mycoparasitism, pathogen, symbiosis

## Abstract

The plant-*Trichoderma*-pathogen triangle is a complicated web of numerous processes. *Trichoderma* spp. are avirulent opportunistic plant symbionts. In addition to being successful plant symbiotic organisms, *Trichoderma* spp. also behave as a low cost, effective and ecofriendly biocontrol agent. They can set themselves up in various patho-systems, have minimal impact on the soil equilibrium and do not impair useful organisms that contribute to the control of pathogens. This symbiotic association in plants leads to the acquisition of plant resistance to pathogens, improves developmental processes and yields and promotes absorption of nutrient and fertilizer use efficiency. Among other biocontrol mechanisms, antibiosis, competition and mycoparasitism are among the main features through which microorganisms, including *Thrichoderma*, react to the presence of other competitive pathogenic organisms, thereby preventing or obstructing their development. Stimulation of every process involves the biosynthesis of targeted metabolites like plant growth regulators, enzymes, siderophores, antibiotics, etc. This review summarizes the biological control activity exerted by *Trichoderma* spp. and sheds light on the recent progress in pinpointing the ecological significance of *Trichoderma* at the biochemical and molecular level in the rhizosphere as well as the benefits of symbiosis to the plant host in terms of physiological and biochemical mechanisms. From an applicative point of view, the evidence provided herein strongly supports the possibility to use *Trichoderma* as a safe, ecofriendly and effective biocontrol agent for different crop species.

## 1. Introduction

It is predicted that by 2050, the world’s overall population will reach 9.1 billion people approximately. Therefore, to feed this increasing world population, a raise of about 70% in agricultural food production is necessary [1]. The substantial increase in food grain production helped in meeting the world food security needs, but problems like global warming, environmental pollution and population explosion has pushed plants towards various kinds of biotic and abiotic stresses which are responsible for yield loss to a large extent and it is an issue of great concern for the wellbeing of our future generations. Biotic stress factors involve fungi, bacteria, virus, nematodes weeds, and insects, which cause a yield loss up to 31–42% [2]. Among them, fungal pathogens are the most severe limiting factor for crop production worldwide. Greater than 10,000 spp. of fungi are considered as responsible for a plethora of plant diseases. Consequently, chemical fungicides are still employed injudiciously as a primary means of disease control. These chemicals are not only expensive, but their application results in the build-up of harmful level of toxins in human beings and in our ecosystem [3,4].

Moreover, the indiscriminate use of fungicides compels the pathogens to undergo genetic mutations which are eventually ascribed to the selection of fungicide resistant biotypes. For instance, *Venturia inequalis* [5], *Phytophthora infestans* [6], *Colletotrichum musae* [7] and *Colletotrichum gloeosporioides, Diplodia natalensis, Phomopsis citri* [8,9] turn resistant to dodine, metalaxyl, benomyl and benzimidazole, respectively. Recently, agronomist and commercial sectors have shown keen interest towards the development of ecofriendly and cost-effective strategies for plant disease management [10].

Biological control mechanisms are contemplated as significant measures for disease management because chemical fungicides adversely affect other non-target organisms [11]. There are several bodies of evidence which support the fact that some microorganisms cause growth inhibition of pathogenic spp. by impairing their metabolisms and/or establishing a parasitic relationship [10]. Additionally, the application of biological control agents (BCAs) with reduced concentrations of chemicals stimulates disease suppression in a similar manner to high doses of chemical fungicide treatments [12]. Around 90% of fungal biocontrol agents against pathogenic microorganisms belong to different strains of *Trichoderma* [13]. *Trichoderma* was isolated for the first time in 1794 from soil and decomposing organic matter [14]. Throughout the world, currently greater than 60% efficacious bio-fungicides are obtained from *Trichoderma* [15]. For example, in India approximately 250 *Trichoderma*-derived bio fungicides products are employed, but in comparison to biological control, Indian farmers are still relying on synthetic chemical fungicides to a greater extent [16].

Different strains of *Trichoderma* (telomorph *Hypocrea)* belong to *fungi imperfecti* as they do not possess any known sexual stage in their life cycle [17]. These fungi are rapid colonizers, invasive, filamentous, opportunistic, avirulent and exhibit a symbiotic relationship with plants. In pathogen-contaminated soils they not only improve plant growth but also inhibit pathogen growth through several antagonistic mechanisms [18,19,20]. *Trichoderma* exhibit antagonistic behavior against several phytopathogenic organisms, including bacteria, nematodes and especially fungi, by inhibiting their growth either by direct interactions (e.g., hyperparasitism, competition for nutrient and space, and antibiosis) [21] or indirectly by improving plant growth and vigor and enhancing stress tolerance, active uptake of nutrients and bioremediation of contaminated rhizosphere, as well as providing plants several secondary metabolites, enzymes and PR proteins [22].

## 2. *Trichoderma*-Plants Interactions

**Figure 1:**
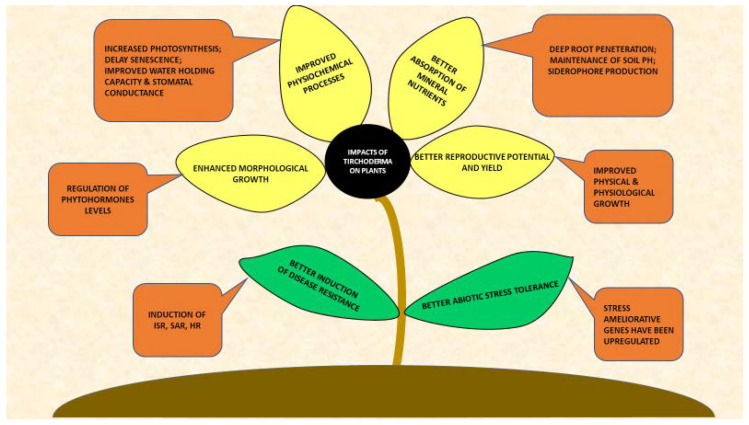
Depicts pictorially the impacts of *Trichoderma* spp. on plants in rhizosphere. Presence of *Trichoderma* improved the plant growth and development at physiological and biochemical levels. Further, *Trichoderma* spp. raised the plant resistance towards several biotic as well as abiotic stresses through multiple adaptive mechanisms.

In recent years, *Trichoderma* has acquired high importance because of its fungicidal and fertilizing potential. In exchange for sucrose from plants, fungi exert numerous advantageous influences on plants. Among them should be mentioned the induction of rapid plant development and production, an increase in nutrient absorption, rhizosphere modification and tolerance improvement to both biotic and abiotic stresses (Figure 1) [13,20,23]. *Trichoderma* is attracted by chemical signals released by a plant’s root. The initial steps of symbiosis establishment involve attachment and penetration and colonization of *Trichoderma* within the plant roots. Plant root anchoring is facilitated by cysteine-rich proteins known as hydrophobin, e.g., TasHyd1 and Qid74 hydrophobins were obtained from *T. asperellum* and *T. harzianum,* respectively [24,25]. After successful attachment, root invasion is promoted by emission of expansin-like proteins. They exhibit cellulose binding modules as well as express endopolygalacturonase activity [26,27]. Furthermore, successful penetration of *Trichoderma* is followed by a rapid colonization of root tissues, which is achieved by lowering plant defenses, such as phytoalexin production, as previously observed in *Lotus japonicus* roots during *T. koningii* penetrations [28]. Moreover, in pathogen contaminated soil, *Trichoderma* spp. cooperate with other beneficial microbial populations, improving plant growth and survival [29,30].

### 2.1. Impacts on Plant Morphology

A lot of evidence indicates that the application of *Trichoderma* spp. to plant rhizosphere promotes plant morphological traits such as root-shoot length, biomass, height, number of leaves, tillers, branches, fruits, etc. [31,32]. For instance, inoculation of soil with *T. atrovirde* enhanced root hair numbers as well as lateral roots in *A. thaliana* [33]. Similarly, application of *T. harzianum* to cucumber roots increased biomass [34] and lateral root formation [35]. Likewise, application of *T. longipile* and *T. tomentosum* significantly enhanced the total leaf area as well as fresh weight in cabbage seedlings as compared to untreated plants grown in a greenhouse [36].

### 2.2. Impacts on Plant Physiology

It has been proven that *Trichoderma* spp. positively regulates several physiological processes in plants such as photosynthesis, stomatal conductance, gas exchange, nutrient absorption and assimilation, water use efficiency, etc. As previously described, *Trichoderma* spp. improved both root growth and the uptake of mineral nutrients from soil. *Trchoderma* spp. treatment significantly improved Mg uptake, a key chlorophyll constituent also involved in catalyzing enzymatic activity as well as in regulating genes engaged in photosynthesis. Moreover, in rice plants treated with *Trichoderma*, the photosynthetic rate (three-folds), stomatal conductance (three-folds) and water use efficiency (two-folds) were significantly stimulated in comparison to plants treated with the classical NPK (Nitrogen, Phosphorus and Potassium) fertilization [37]. In addition, treatment of rice plants with *T. harzianum* increased water holding capacity, enhanced drought stress resistance and delayed plant senescence phenomenon [38]. A similar senescence delay was observed in rice after application of *Trichoderma* spp. [39].

### 2.3. Impacts on Nutrient Solubilization and Absorption

Roots of *Trichoderma*-treated plants have exhibited a higher ability to explore the soil and an improved uptake of minerals. According to Harman et al. [40] different strains of *Trichoderma* emit several acids such as coumaric, glucuronic and citric acids, which assist in the discharge of phosphorus ions, which seem to be inaccessible to plants in most soils [41]. The presence of *T. harzianum* strain 1295-22 in soil increases the availability of P as well as Fe and Zn in liquid medium [42]. Similarly, application of strain T-203, also known as *T. asperelloides*, enhanced the available amount of Fe and P in the rhizosphere to an amount of 30% and 90%, respectively. Moreover, root and shoot growth, in response to *Trichoderma* inoculation, leads to an increase of Cu, Na and Zn uptake as well as other micronutrients [43]. Iron deficiency in alkaline soil is a major drawback for crop production in agriculture. The potential ability of *Trichoderma* for siderophore production can be used to cope with this problem. It has been reported that the application of *T. asperellum* (T-6) to cucumber roots increased Fe^2+^ and siderophore content in soil as well as the activity of Fe^2+^ and Fe^3+^ chelate reductase [40]. Furthermore, [44] Colla et al. [44] reported that two kinds of siderophores (hydroxamate and catechol) were produced by the MUCL45632 strain of *T. atroviride.* These studies highlight that *Trichoderma* application in soil assists the plant in reduction of Fe^3+^ to Fe^2+^, which consequently boosts its solubilization and uptake.

### 2.4. Yield Improvement

Treatment with different species of *Trichoderma* guarantees high yield production in the case of crops like mustard, wheat, corn, tuberose, sugarcane, tomato, okra, etc. [45,46,47,48,49,50]. Similarly, seed biopriming with *Trichoderma* spp. spores substantially improve crop yield in greenhouses conditions [51]. Likewise, *T. harzianum* and *T. viride* treatments applied to marigold, petunia and verbena induced a significant increase in the number and weight of the flowers [52]. Moreover, treatment of chili seeds with *T. harzianum* IMI-3924332 enhances the germination rate [53].

### 2.5. Impacts on Abiotic Stress Tolerance

Being sessile organisms, plants are frequently exposed to various abiotic stresses. Inoculation of soil with different strains of *Trichoderma* improves plant growth and reproduction under stressful conditions. For example, biopriming of rice with *T. harzianum* reduced the harmful effects of salinity stress on plants and improved the plant growth [54]. Similar findings were also obtained in plants exposed to salinity stress, e.g., *T. asperellum* Q1-treated cucumber [55] and seedlings of *Arabidopsis thaliana* remedied with *T. asperelloides* T203 [56]. During heat and cold stresses, *Trichoderma* spp. also play a crucial role in their mitigation. For example, chilling stress in tomato plants was mitigated when plants were treated with *T. harzianum* AK20G strains [57]. Similarly, transgenic plants of *A. thaliana* exhibited a greater tolerance to heat stress when transformed with *T. harzianum* T34 *hsp70* genes [58]. Furthermore, various species of *Trichoderma* are also known for their roles in amelioration of oxidative stress in plants. In fact, in wheat plants inoculated with *T. longibrachiatum* and subjected to salinity, a significant increase in antioxidants like SOD (superoxide dismutase), CAT (catalase) and POD (peroxidase) gene expression was observed [59].

### 2.6. Induction of Disease Resistance

It has been reported that the addition of different species of *Trichoderma* in a plant’s rhizosphere improved plant defense against several pathogenic organisms such as viruses, bacteria and fungi, by stimulating the initiation of different resistance mechanisms mainly encompassing induced systemic resistance (ISR), hypersensitive response (HR) and systemic acquired resistance (SAR) [40]. Based on several reports (Table 1), an inference in favor of different classes of metabolites can be outlined, which emphasizes their significance as elicitors or resistance inducers in the *Trichoderma*-plants interactions [60]. These metabolites incorporate proteins displaying enzymatic activity such as xylanases and chitinases, protein-like gene products expressed by non-virulent genes and low molecular composites produced because of hydrolytic enzymatic degradation of fungal or plant cells [60].

Induction of resistance is due to the rise in the amounts of defensive metabolites as well as enzymes. These mainly include phytoalexin biosynthesis (HR), which involves the participation of enzymes of phenylpropanoid metabolism, i.e., phenylalanine ammonialyase (PAL) and chalcone synthase (CHS) [61]. Other enzymes which enhance resistance in plants also include chitinases and glucanases [62]. They also encompass pathogenesis-related proteins (PR) (SAR response), and enzymes play a part in antioxidative defense response [61]. For example, *Hordeum* spp., exhibiting *Trichoderma* *atroviride* endochitinase Ech42 activity, revealed improved resistance for *Fusarium* infection [62]. Likewise, *T. harzianum*-derived chitinase (Chit42), expressed in tobacco and potato plants, led to the development of extremely tolerant or totally resistant transgenic lines towards soil-borne pathogen like *Rhizoctonia solani* as well as foliar pathogens such as *Alternaria alternata, A. solani* and *Botrytis cinerea* [63]. Yedidia et al. [64] confirmed that cucumber roots inoculated with *T. harzianum* were characterized by a higher expression of peroxidase and chitinase activities, which improved plant resistance to pathogenic attacks.

## 3. *Trichoderma*-Pathogen Interactions

Disease control, as facilitated by biocontrol mediators, is an outcome of the interactions among the plant’s symbiont and pathogenic communities. Because of their capability to defend plants and control pathogen populations, under various soil circumstances, *Trichoderma* spp. have been extensively analyzed and exploited commercially as biocontrol agents, soil improvers and biofertilizers, placing *Trichoderma* spp. amongst the most explored fungal BCAs [20,40,65]. Several species of this genus are ‘rhizosphere competent’ and can also decompose polysaccharides, hydrocarbons, chlorophenolic compounds and the xenobiotic pesticides employed in cultivation [66]. The key biocontrol strategies that *Trichoderma* develops in direct conflict with fungal pathogens are mycoparasitism [67,68], competition [60] and antibiosis [69,70].

### 3.1. Mycoparasitism

Mycoparasitism implies the direct strike of one fungal species on another and is among the most important antagonistic mechanisms expressed by *Trichoderma* spp. About 75 *Hypocrea/Trichoderma* species with mycoparasitic potential have been previously reported. There are several investigations which indicate that numerous strains of *Trichoderma* attack and disintegrate plant pathogenic fungi, e.g., *Rhizoctonia solani*, *Alternaria alternata*, *Sclerotinia sclerotiorum*, *Fusarium* spp., *Botrytis cinerea, Pythium* spp. and *Ustilago maydis* [40,70,71].

About 70 years ago, Weindling [72] was the first to note this mycoparasitic reaction. This complex process includes sequential events. Firstly, identification between *Trichoderma* and the target fungus is mediated by the binding of carbohydrates present in the cell wall of *Trichoderma* to the lectins of the other one. This is followed by the hyphal twirling and appresoria development, which encompasses a greater number of osmotic compounds like glycerol. After successful penetration, *Trichoderma* initiate the attack on the host’s cellular machinery via generating numerous fungitoxic cell wall degrading enzymes (CWDEs), such as glucanases, chitinases and proteases [40]. The cumulative action of these compounds causes dissolution of the host cell walls, which ultimately results in parasitism of the target fungus. It has been observed that gaps can be generated at the location of appressoria formation which facilitate the direct access of *Trichoderma* hyphae into the lumen of the target fungus, which then proceeds to kill the pathogenic fungus [22]. Furthermore, biocontrol agents not only degrade the cell wall of target fungus, but also inactivate its enzymes (e.g., pectinases etc.), which are essential for pathogenic fungus to colonize and penetrate the plant tissues [40].

As we know, fungal cell walls are mainly composed of chitin and β-1,3-glucan [73]. Chitinases (EC 3.2.1.14) and β-1,3-glucanases (EC 3.2.1.39) lytic enzymes synthesized by *Trichoderma* spp. are supposed to be responsible for their mycoparasitic actions leading to the degradation of phytopathogenic fungal cell walls [74,75,76]. In addition, other CWDEs including those hydrolyzing minor polymers (like proteins, β-1,6-glucans, α-1,3-glucans, etc.) further ensure the complete and effective disintegration of fungal mycelial or conidial walls by *Trichoderma* spp. [77]. A chitin induced subtilisin-type serine proteinase has previously been depicted in a *Trichoderma harzianum* mycoparasitic strain [76]. Moreover, β-1,6-glucanases (EC 3.2.1.75) have been reported to degrade cell walls in yeast, filamentous fungi [78,79] and bacteria [80] (Table 1).

Zeilinger et al. [81] previously reported that *Trichoderma* can sense the existence of pathogenic mycelium in the rhizosphere and proliferate towards the direction of the pathogen area. Recently, the green fluorescent protein encoding gene was incorporated downstream to the regulatory sequence of an endo- and an exochitinase encoding gene. This study revealed that, during the *Trichoderma*-fungal interaction, the endochitinase gene is stimulated prior to contact with the target fungus. On the contrary, exochitinase activation took place only after the contact was established [82]. Distinct forms may pursue separate patterns of stimulation, however, *Trichoderma* in fact constantly emit small amounts of exochitinase. Transmission of this enzyme stimulates the generation of cell wall pieces from target fungi. These fragments apparently interact with receptors on the cell wall or plasma membrane of *Trichoderma* and consequently promote the expression of fungitoxic CWDEs [83]. These CWDEs in turn diffuse and initiate the attack on the target fungi before the actual contact has been made [80,84]. As soon as the contact has been established, *Trichoderma* spp. coil and form appressoria on the exterior of the host. In addition to CWDEs, *Trichoderma* emits fungitoxic peptaibol antibiotics [85]. The collective action of these ingredients is essential for dissolution of the cell walls and parasitism of the target fungus. Approximately 20–30 known genes, proteins or metabolites are clearly engaged in this activity [86,87].

### 3.2. Competition

The limited availability of and competition for nutrients lead to the natural management of fungal communities and phytopathogen development [51]. Competition for micro- and macronutrients such as C, N and Fe plays a pivotal role during interactions of advantageous and disadvantageous fungi and is coupled with the biocontrol systems [18]. It has been well established that *Trichoderma* species compete for nutrients, biological niches or infection spots with pathogens in plant rhizosphere [60]. *Trichoderma* exhibits a better capability to mobilize and absorb nutrients from the soil in comparison to other rhizospheric microorganisms; therefore, the control management of some pathogens (e.g., *B. cinerea*) by using *Trichoderma* involves the coordination of numerous strategies, such as the competition for nutrients, which is considered amongst the most important [88].

The effective utilization of nutrients depends upon the ability of *Trichoderma* spp. to get energy derived from the metabolism of carbohydrates like cellulose, chitin, glucan and glucose, which are often present in the mycelial environment [51]. The function of the glucose transport system has yet to be discovered, but it is conceivable that its competence in *Trichoderma* competition performs a pivotal role [89]. Root exudates and the rhizosphere are particularly rich in nutrients like carbohydrates, amino acids, organic acids, vitamins, Fe, etc., but the competition for C between *Trichoderma* and pathogenic fungi like *Rhizoctonia solani*, *F. oxysporium*, etc. was considered to be most noteworthy [90,91].

As compared to other microbes in the soil, the competent mobilization of immobile nutrients and their use provides superiority to *Trichoderma.* For this purpose, *Trichoderma* induces the reduction of soil pH via the biosynthesis and release of organic acids like gluconic, citric and fumaric. Moreover, these organic acids further facilitate the solubilization of micronutrients and mineral cations such as phosphates, Fe, Mn and Mg [18]. Interestingly, it has been reported that *T. harzianum* CECT 2413 encodes a glucose transporter (Gtt1) which expresses a high affinity for glucose even at an exceptionally low concentration [89,92]. Moreover, Vargas et al. [93] recognized an intracellular invertase enzyme from *T. virens* (TvInv) which seems to be responsible for the degradation of plant-derived sucrose.

**Figure 2:**
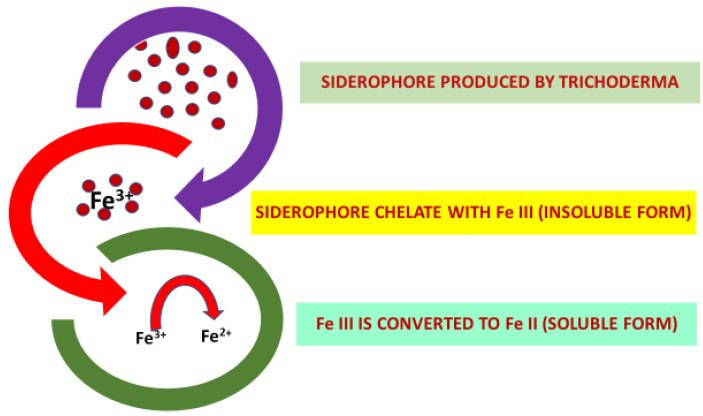
In plant rhizosphere *Trichoderma* produces a siderophore which chelates insoluble Fe (Fe^3+^) and facilitate its conversion to soluble Fe (Fe^2+^) form. By doing this, *Trichoderma* also make Fe source unavailable to pathogenic fungi and thereby deprive them of Fe.

Fe ions serve as cofactor for multiple classes of enzymes and play a key role as a nutrient for the growth and development of plants [94]. Iron occurs primarily as Fe^3+^ under the conditions of neutral pH and in the presence of oxygen. In the aerobic environment, Fe tends to develop insoluble ferric oxide, which ultimately makes it not available for root absorption [94]. A Fe-chelating complex, known as siderophore, is secreted by *Trichoderma* spp. [95]. This complex first binds to the insoluble iron (Fe^3+^) and then transforms it into the easily absorbable soluble form, i.e., (Fe^2+^) (Figure 2). While increasing the availability of Fe to plants, siderophore simultaneously depletes the Fe sources of the soil and thereby inhibits the growth of target fungi [95]. Most of the fungal siderophores derived so far relate to the hydroxamate class and can be classified into three families: fusarinines, coprogens and ferrichromes [96,97].

### 3.3. Antibiosis

Antibiosis is the process by which diffusible low-molecular weight compounds interact and reduce the growth of other microorganisms. Mainly, antibiosis is centered on the production of secondary metabolites, which display an inhibitory or deadly consequence on a parasitic fungus. More than 180 secondary metabolites indicating distinct classes of chemical products have been isolated from fungal species belonging to genus *Trichoderma* [98,99]. Depending upon their biosynthetic origins, these compounds can be grouped into peptaibol, polyketide and terpene [100]. Various spp. of *Trichoderma* are known to produce non-proteinogenic amino acid (especially α-aminoisobutyric) composed peptaibols, which are polypeptide antibiotics with a molecular weight ranging from 500 to 2200 Da. The peculiar feature of these compounds is that their N-terminal is acetylated, while the C-terminal has amino alcohols [101]. Therefore, their chemical nature is amphipathic, and they arrange themselves in the membrane to form voltage-gated ion channels. These peptides are synthesized by non-ribosomal peptide synthetases (NRPSs).

In addition to this, *Trichoderma* spp. express the capability to synthesize a different class of defensive metabolite, termed polyketides, through sequential events catalyzed by a complex of enzymes called as polyketide synthases (PKSs). Different strains of *Trichoderma synthesize* a huge variety of antibiotics [99], e.g., *T. viride* produces trichotoxins A and B, trichodecenins, trichorovins and trichocellins. Similarly, trichorzianins A and B, trichorzins, HA and MA were isolated from culture filtrate of *T. harzianum*. *T. longibrachiatum* produces tricholongins BI and BII, whereas longibrachins and trichokonins were isolated from *T. koningii*; atroviridins A-C and neoatroviridins A-D derive from *T. atroviride* cultures. Further, other antibacterial and fungicidal metabolites, e.g., koningins, viridin, dermadin, trichoviridin, lignoren and koningic acid were isolated from *T. koningii*, *T. harzianum*, *T. aureoviride*, *T. viride*, *T. virens*, *T. hamatum* and *T. lignorum* cultures [99]. Gliotoxin and gliovirin are among the most significant secondary metabolites of *Trichoderma* related to the P and Q group strain, respectively (Table 1). P group strains of *Trichoderma* (*Gliocladium*) *virens* adversely affect *P. ultimum*, but not *R. solani.* On the other hand, Q group is more active against *R. solani* [102]. The *T. virens* gene *ve*A ortholog *vel*1 encoded the VELVET protein, which regulates both the biosynthesis and the biocontrol activity of gliotoxin as well as other genes participating in the secondary metabolism [103].

Growth of soil-borne pathogens like *R. solani, Phytophthora cinnamomi, Pythium middletonii, Fusarium oxysporum* and *Bipolaris sorokiniana* was observed to be negatively affected in the presence of Koninginin D [104]. In a similar way, viridins obtained from *Trichoderma* spp. like *T. koningii, T. viride,* and *T. virens* contained the spore germination of *Botrytis allii, Colletotrichum lini, Fusarium caeruleum, Penicillium expansum, Aspergillus niger* and *Stachybotrys atra* [105]. *T. harzianum*-derived harzianic acid showed antibiotic activity against *Pythium irregulare, Sclerotinia sclerotiorum* and *R. solani* in in-vitro culture [106]. Two asperelines (i.e., A and E) and 5 trichotoxins designated as T5D2, T5E, T5F, T5G and 1717A with antibiotic features were produced by the *T. asperellum* strain [107]. In general, antibiotic activity is combined cooperatively with lytic enzymes. Their dual action offers a more advanced level of antagonism than the activity of either antibiotics or enzymes acting alone [108]. As observed by Howell et al. [63], initial disintegration of cell walls in the case of *B. cinerea* and *F. oxysporum* by lytic enzymes enhanced the antibiotic penetration into the target hypha.

## 4. Effect of *Trichoderma* Inoculation

### 4.1. Destruction of Pathogenic Organism

**Figure 3:**
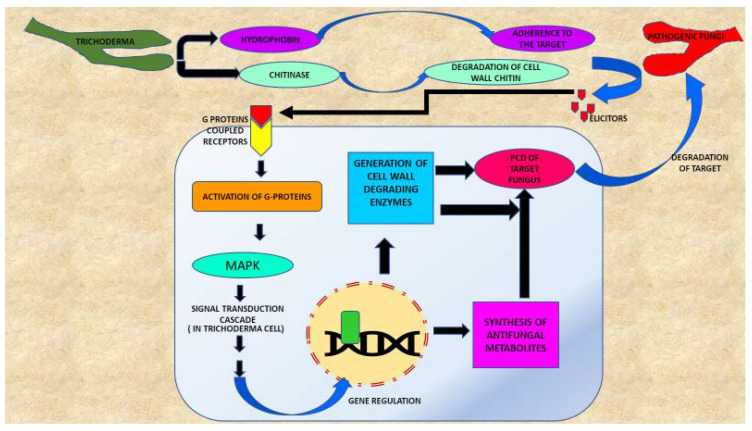
Mode of action of *Trichoderma* spp. in destroying pathogenic fungi. *Trichoderma* releases the lytic enzymes in the rhizosphere, which catalyzes the cell wall damage to target fungi. After this, a signaling cascade is activated in *Trichoderma* cells which involves the activation of MAPK (mitogen-activated protein kinase) through G-protein-coupled receptors. Alteration in gene expression ultimately leads to PCD (programmed cell death) of pathogenic fungi.

This complex process includes sequential events, which initially involve recognition between *Trichoderma* and the target fungus, the coiling around the fungal hyphae, which is followed by appresoria development [40]. After this collective action, lytic enzymes cause the dissolution of target fungal cell walls. Furthermore, *Vel1* of *Trichoderma virens* participates in the expression of hydrophobin, which facilitates the adhesion of *Trichoderma* to the host [24]. Interestingly, seven transmembrane G protein coupled receptors (*Gpr1*) are engaged in perceiving the target fungus in the adjacent neighborhood [109,110]. Binding of ligands with such receptors causes the downstream signaling cascade via stimulation of G proteins and mitogen-activated protein kinase (MAPK). Three MAPK (i.e., MAPKKK, MAPKK and MAPK) are known in different species of *Trichoderma* [111]. These signaling pathways might play an important role during mycoparasitism and biocontrol of pathogens [111,112] (Table 1). Manufacture and discharge of CWDEs and antibiotics are extremely valuable members of the chemical resources used by *Trichoderma* to eradicate the pathogens (Figure 3).

*Trichoderma* also owns glucan and chitin synthases, which are enzymes involved in the healing of the *Trichoderma* cell wall, which might be damaged during *Trichoderma*–pathogen contact. Simultaneously, hydrolytic enzymes like chitinases and glucanases, as well as those for secondary metabolism like the NRPSs (non-ribosomal peptide synthetases) pathway, are expressed, inducing pathogen death [98]. Participation of *chit42*, *chit3*, *bgn13.1*, *Bgn2*, *Bgn3* and *prb1* genes in biocontrol of deleterious fungi through the activities of chitinases, glucanases and proteases were demonstrated [113].

Certain *Trichoderma* species (e.g., *T. atroviride*) produce 6-pentyl-2H-pyran-2-one (6-PP), a volatile metabolite which plays a key role during *Trichoderma*–fungal interactions [106,114]. Recently, genetic investigations unveiled that NRPS Tex2 of *T. virens* causes the assemblage of 11- and 14-module peptaibols [115], and these peptaibiotics strongly exhibit antimicrobial activities. For instance, a *T. pseudokoningii* peptaibol, called trichokonin VI, is known to form voltage-gated channels in membrane, and it ultimately induces programmed cell death (PCD) in *Fusarium oxysporum* [116]. Similarly, trichokonins VI, a peptaibol isolated from *T. pseudokoningii* SMF2, displays antibiotic actions by stimulating wide-ranging apoptotic PCD in a range of fungal pathogen species [117]. In a mutant of *T. brevicompactum,* namely *Tb41tri5,* the promoted expression of the *tri5* (*trichodiene synthase*) gene amplified the synthesis of trichodermin. Additionally, it enhanced the antifungal activity against *Aspergillus fumigatus* and *Fusarium* spp. [115,117].

### 4.2. Plant Growth Promotion

Root colonization by *Trichoderma* in both mono- and dicotyledonous plants might cause noteworthy variations in plant metabolism. These mainly include alteration in the biosynthesis of growth regulators, compatible osmolytes, amino acids and phenolic components, as well as other physiological processes like photosynthesis, transpiration and leaf water potential [118,119]. Many lytic enzymes such as cellulase, xylanase, pectinase, endopolygalacturonase, glucanase, lipase, amylase, arabinase and protease have been isolated from different strains of *Trichoderma* [120,121]. A cellulose-binding protein termed swollenin can disrupt the crystalline structure of cellulose in plant cell walls [26]. It possesses a sequence similarity with plant protein expansins, which simplifies expansion of the plant cell wall in roots, as well as in root hairs. Via swollenin production, *Trichoderma* may enhance the surface area of plant roots, improving its establishment in the rhizosphere [26,70].

**Figure 4:**
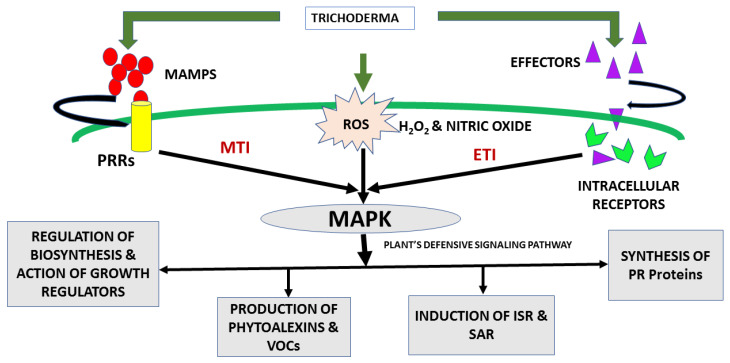
Plant-*Trichoderma* interaction involves the recognition molecules, i.e., MAMPS (microbe-associated molecular patterns) and effectors. MAMPS and effector molecules bind to the PRRs (pattern recognition receptors) and intracellular receptors and thereby initiate the MTI (MAMPS triggered) and ETI (effector triggered) immunity in plants, respectively. Moreover, this interaction also leads to the production of ROS (reactive oxygen species), which serve as signaling molecules and initiate a defensive response in plants by synthesis of antifungal molecules like phytoalexins, VOCs (volatile organic compounds), PRs (pathogenesis related) proteins such as CWDEs, etc. *Trichoderma* also improved the plant growth in pathogen-contaminated soil by regulating the expression of genes involved in growth regulation as well as induction of disease resistance.

In general, an immune-like system is exhibited by plants which has the potential to perceive domains/motifs with preserved structural characters distinctive of a family of microbes termed as microbe-associated molecular patterns (MAMPs) (Figure 4) [13]. The ability of *Trichoderma* spp. hyphae to release MAMPs for molecular recognition may contribute to signal cascade by signaling molecules within the plant. *Trichoderma* acts locally and systemically, involving signaling cascade and activation as well as accumulation of defense-related antimicrobial compounds and enzymes such as phenyl ammonia lyase (PAL), peroxidase, polyphenol oxidase and lipoxygenase. In addition, PR proteins, terpenoid, phytoalexins (rishitin, lubimin, phytotuberol, coumarin, solevetivone, resveratrol, etc.) and antioxidants (ascorbic acid, glutathione, etc.) are also synthetized [102]. Consequent upon fungal invasion, plants respond to *Trichoderma* colonization by producing and concentrating defensive compounds like phytoalexins, flavonoids, terpenoids, phenolic byproducts, aglycones and additional antimicrobial compounds. Interestingly, *Trichoderma* strains are normally resistant to such compounds. This resistance is regarded as a crucial prerequisite to colonize the plant roots, and it has mainly been contributed by ABC (ATP-binding cassette) transport systems present in *Trichoderma* strains [122].

Reactive oxygen species (ROS) like H_2_O_2_, nitric oxide, etc., produced by glucose oxidase enzymes, are linked to *Trichoderma*-intermediated immunity in cotton, rice and *A. thaliana* [123,124,125]. Defense signaling in plants involves the participation of mitogen-activated protein (MAP) kinases, which convey information from receptors to initiate a cascade of cellular responses in plants (Figure 4) [126]. As reported in the case of cucumber, a MAPK exhibiting similarity with MPK3 of *A. thaliana* is stimulated via inoculation of the root with *T. asperellum* [127]. In a similar manner, an increase of concentration of the phytoalexin camalexin was detected in the *T. virens-* and *T. atroviride-*colonized root system of *A. thaliana* [128].

Molecular studies in *A. thaliana* revealed that colonization of roots by *T. asperelloides* T203 activated a quick upsurge in transcription factor (*WRKY18*, *WRKY40*, *WRKY60* and *WRKY33)* expression, which further suppresses salicylic acid (SA) signaling and triggers jasmonic acid (JA)-pathway responses. These genes are induced by pathogens and their expression encodes three WRKY structurally linked proteins that play a key role in JA-arbitrated defense [56]. The expression of *PR-1a* (pathogenesis-related) and SA regulated genes, as well as the *LOX2* gene, were upregulated by the application of *T. atroviride* and *T. virens* to *A. thaliana* [128,129]. Moreover, *T. harzianum* amplified the levels of SA and JA in melon and thereby changed the plant reactions against *F. oxysporum* [130]. Likewise, expression of *LOX* and *PAL1* genes (involved respectively in the biosynthesis of jasmonic acid and salicylic acid) and *ETR1* and *CTR1* genes (participating in ethylene signaling pathways) were observed to increase after the application of *T. asperellum* T203 [131] (Table 1).

Cellulysin, isolated from *T. viride,* stimulates the octadecanoid signaling pathway, which subsequently activates the discharge of several volatile compounds in plants [132]. As reported in the case of leaves of lima bean, cellulysin together with JA induce the synthesis of dimethyl nonatriene, hexenyl acetate, germacrene, ocimene, caryophyllene and copaene. Another resemblance between JA- and cellulysin-induced actions causes the discharge of ethylene [132]. Beside degradation of xylan, *β*-1,4- endoxylanase (EIX) activity from *T. viride* provoked ethylene emission and the plant defensive system in tobacco [133]. A rise in ethylene levels is supplemented by buildup of ACC (1-aminocyclopropane-1-carboxylic acid) due to enhancement in ACC synthase activity as well as increase in ACC oxidase transcripts [134]. In addition, it has been observed in rice plants that EIX behaved as fungal elicitors, controlling phytoalexin biosynthesis and the expression of defensive genes via calcineurin B-like protein-interacting protein kinases (OsCIPK14/15) [135]. Similarly, SM1, a fungal elicitor obtained from *T. virens,* encourages the expression of the *CAD1- C* gene in cotton petioles, which encodes the enzyme (+)-*δ*-cadinene synthase. This enzyme serves as a primary inducer for phytoalexin synthesis in response to pathogen invasion [122,136].

**Table 1:** Compounds synthesized by *Trichoderma* spp. involved in plant interaction.

Sr. No.	Category	Sub-Category	Function Performed	*Trichoderma* Species	References
1.	**Phytohormones**				
IAA			Growth and development of plants and their root system.	*T. virens*	[35]
GA_3_			Growth promotion by degradation of growth repressing DELLA proteins and reduction in ethylene level.	*Trichoderma* spp.	[13,137]
ABA			Alteration in transpiration and regulation of stomatal aperture via induction of an ABA receptor.	*T. virens and T. atroviride*	[33]
Ethylene			Improved tolerance to biotic as well as abiotic stresses by regulation of levels of SA and JA as well as their signaling pathways.	*T. atroviride*	[138,139,140]
JA			JA and/or ET are the signaling molecule for *Tichoderma*-induced ISR.	*T. asperellum*	[141]
SA			Enhances disease resistance in plants through induction of SAR.	*T. atroviride*	[26,142,143]
2.	**Enzymes**				
		**Hydrolytic**			
Cellulolytic enzymes			Cleavage of β-1,4-D-glycosidic bonds in cellulose molecule.		[120]
Exo-β-1,4-glucanases			Breakdown of cellulose by forming a cellobiose molecule either from the reducing or nonreducing terminals.	*T. viride*, *T. harzianum*, *T. reesei*, *T. koningii*	[144]
Endo-β-1,4-glucanases			At the time of enzymatic lysis of cellulose, break the β-1,4- glycosidic bonds in a random way probably in the amorphous areas of cellulose and thereby cause formation of cellulodextrines with variable chain lengths.	*T. viride*, *T. longibrachiatum*, *T. pseudokoningii and T. reesei*	[145,146,147]
β-Glucosidases			Promote lysis of short length oligosaccharides and cellobiose into glucose.	*T. viride*, *T. harzianum*, *T. reesei and T. longibrachiatum.*	[148,149]
Xylanase			Catalyze breakdown of xylans to form xylo-oligomers, xylobiose and xylose.	*T. harzianum*, *T. koningii*, *T. lignorum*, *T. longibrachiatum*, *T. pseudokoningii*, *T. reesei*, *T. viride* *Trichoderma harzianum*, *T. virens*, *T. asperellum*, *T. atroviride*	[150]
Chitinase			Catalyze degradation of chitin to chitooligomers of low molecular weight.		[83,151,152,153,154]
Endochitinases			Randomly hydrolyses chitin at internal sites and form dimer of diacetylchitobiose and low molecular weight multimers of GlcNAc like chitotriose and chitotetraose.		
Exochitinases			Divided into 2 subcategories: 1. Chitobiosidases, involved in catalyzing the sequential release of diacetylchitobiose starting from the non-reducing end of the chitin microfibril 2. 1-4-β-glucosaminidases, splitting the oligomeric products of endochitinases and chitobiosidases, thereby producing GlcNAc monomers.		
		**Proteases**			
Exopeptidases			Cause the cleaving of peptide bond either at the amino or carboxy terminal.	*T. viride*, *T. harzianum*, *T. aureoviride*, *T. atroviride*	[155,156]
Endopeptidases			Split the peptide bonds away from the ends.		
Lipase			Lipase hydrolyses ester bonds of triacylglycerols, resulting in the formation of mono- and diacylglycerols, free fatty acids and, in some cases, glycerol also.	*T. lanuginosus*, *Trichoderma reesei*, *Trichoderma koningii*, *T. harzianum*, *T. virens*, *m T. viride*	[157]
Glucose oxidase			Cause generation of reactive oxygen species (ROS).	*T. virens*, *T. asperelloides*	[123,124,125]
Antioxidative enzymes (e.g., SOD, CAT, POD etc.)			Enhance antioxidative defense mechanism in plants.	*Trichoderma* spp.	[59,158]
	**Biosynthetic and signaling**				
PAL & CHS			Production of phytoalexins.	*Trichoderma* spp.	[60]
Glucan and Chitin synthases			Produced by the *Trichoderma* to repair their self-cell wall damage by pathogen during *Trichoderma*–pathogen interaction.	*Trichoderma* spp.	[159]
MAPK			Convey information from receptor to generate cellular signaling and defense responses.	*Trichoderma* spp.	[126,131]
ETR1 and CTR1			Involved in ethylene (ET) signaling.	*Trichoderma* spp.	[131]
LOX1 (Lipoxygenase 1) PAL1 (phenylalanine ammonia lyase),			Participate in jasmonic acid (JA) biosynthetic pathway. Involved in biosynthetic pathway for salicylic acid (SA)	*Trichoderma* spp.	[160]
ACC synthase ACC oxidase			Promote ethylene biosynthesis.	*Trichoderma* spp.	[134]
*δ*-cadinene synthase			Act as precursor for phytoalexin synthesis.	*T. virens*	[123,136]
3.	**Soil modifiers**				
Gluconic, citric and fumaric acids			Reduce the pH of soil and facilitate the solubilization of phosphates and micronutrients.	*Trichoderma* spp.	[18,41]
Siderophore			Chelate with insoluble Fe (III) and convert them to soluble Fe (II).	*Trichoderma* spp.	[44,94,95]
4.	**Secondary metabolites**				
Pyrones			Antimicrobial	*Trichoderma* spp.	[161]
Lactones			Participate in IAA and ethylene-mediated signaling and improve plant growth and root architecture.	*T. harzianum*, *Trichoderma cremeum*	[162]
Koninginins			Antimicrobial	*T. koningii*, *T. harzianum*, *T. aureoviride*	[163,164]
Trichodermamides			Antifungal and exhibit cytotoxicity to human colon carcinoma.	*T. virens*	[165,166]
Viridins			Antifungal	*Trichoderma virens*, *T. koningii*, *T. viride*	[99,167,168]
Nitrogen heterocyclic compounds (harzianopyridone, harzianic acid)			Antifungal	*T. harzianum*	[169,170,171]
Azaphilones			Antifungal	*T. harzianum T22*	[171,172]
Butenolides and hydroxy-Lactones (cerinolactone, trichosordarin A, harzianol A and harzianone)			Antifungal	*T. cerinum*, *Trichoderma cremeum*, Trichoderma longibrachiatum A-WH-20-2	[163,173,174]
Isocyano metabolites (dermadin and trichoviridin)			Antifungal	*T. viride T. koningii and T. hamatum*	[164,175,176]
Diketopiperazines (gliotoxin and gliovirin)			Antifungal	*Trichoderma (Gliocladium) virens*	[177]
Peptaibol (alamethicin, trichokonin VI)			Non-ribosomal short peptides, rich in 2-amino-isobutyric acid involved in plant defense and antimicrobial in nature.	*T. virens*, *T. longibrachiatum*	[178,179]
Polyketides			Participate in SA mediated signaling pathway and exhibit antimicrobial activities.	*T. virens*, *Trichoderma* sp. SCSIO41004	[180,181]
Terpenes cyclonerane sesquiterpenoids, trichocitrin, trichosordarin A			Antimicrobial	*T. virens*, Trichoderma harzianum P1-4, *Trichoderma citrinoviride* cf-27, Trichoderma harzianum R5	[182,183,184,185]
Volatile organic compounds (VOCs) (trichodiene)			Facilitate the plant-microbe interactions in rhizosphere	*T. arundinaceum*, *T. atroviride*	[186,187,188]
Hydrophobins			Plant growth promotion, signaling and defense	*T. virens* and *T. atroviride*, *T. asperellum*	[189,190]

## 5. Other Applications of *Trichoderma*

Besides the aforementioned roles of *Trichoderma* spp., their extreme versatility in terms of metabolite production makes fungi from the genus *Trichoderma* potentially interesting for different applications, as detailed below.

### 5.1. Bioremediation

Several deleterious organic pollutants like phenols, cyanides and nitrates are frequently degraded via *T. harzianum* [191]. There are several reports which show the involvement of *Trichoderma* spp. strains in detoxification of polycyclic aromatic hydrocarbons (PAHs). Katayama and Matsumura [192] verified the degradative efficacy of *Trichoderma* spp. against several artificial dyes like pentachlorophenol, endosulfan and dichlorodiphenyl trichloroethane (DDT). Capability of immobilized *T. viride* biomass along with cell-free Ca-alginate beads in biosorption of Cr (VI) has already been reported [193]. Similarly, *T. inhamatum* displayed an extraordinary capability to stand and totally reduce Cr (VI) concentrations, playing a significant role in bioremediation of Cr (VI)-contaminated wastewaters [194]. Likewise, *Trichoderma harzianum* express various adaptive strategies in detoxification of Cd contaminated soil [195].

### 5.2. Animal Feed

Lytic enzymes, like cellulases, hemicellulases and pectinases, produced by *Trichoderma* spp. can be employed in partial hydrolysis of plant cell walls in feeds. This process increases the digestibility of the feed and increases its nutritive value. Therefore, an increase in animal body weight as well as a higher milk yield was observed [196].

### 5.3. Industrial Applications

Cellulases produced by *Trichoderma* are also used to soften textiles. Moreover, the enzymes attained from *Trichoderma* are employed to modify fiber properties as well as to reduce lignin contents [197]. *T. harzianum-*derived mutanase may be added in toothpaste to avoid the development of plaque [198]. In the food industry, additional metabolites obtained from the different species of *Trichoderma* are also used along with their enzymes. For example, nut aroma producing compounds, obtained initially from *T. viride* and afterward from *T. atroviride*, express useful antibiotic properties [199]. Brewery industries also use the enzymes attained from *Trichoderma* spp. They may also be employed as food additives and escalate maceration of raw materials for the manufacturing of fruit and vegetable juices. These enzymes can also be employed to improve wine tang and increase the fermentation, filtration and excellence of beer. Above all, the potential of *Trichoderma*-derived bioactive compounds could be exploited in the pharmaceutical industry because of their several curative properties [200,201,202,203].

### 5.4. Second Generation Biofuels

Improved conservational understanding of whole communities as well as growing concerns in alternative resources of energy make it feasible to use fungi from the genus *Trichoderma* in the manufacturing of self-styled second-generation biofuels [204]. For instance, cellulases and hemicellulases supplied by *T. reesei* are used in the production of bioethanol from farm wastes. These enzymes indeed catalyze the biodegradation of substrates to simple sugars, and afterwards, these are exposed to yeast (*Saccharomyces cerevisiae*)-induced fermentation [205,206].

### 5.5. Wood Preservation

Wood preservation by chemicals is relatively cheap and effectively prolongs the service life of wood [207]. By contrast, the toxicity of heavy metals and other chemicals used as wood preservatives are also a matter of serious health and environmental concern [208,209,210,211]. The intense research activities on developing and testing less problematic protective systems demonstrate the urgent need for innovation in this field [212,213,214,215,216,217,218,219,220]. As the antagonistic properties were evolved in competition with other wood destroyers—such as wood-rotting and sap-staining fungi, or other molds—the expectation is justified that the *Trichoderma* isolated from wood does have the ability to effectively inhibit wood-damaging fungi. Interestingly, Ejechi [221] researched the capability of *Trichoderma viride* to prevent the fungal (*Gloeophyllum* sp. and *G. sepiarium)* decay of obeche (*Triplochiton sceleroxylon*) wood via deterioration of decaying fungi under field conditions. Similarly, Tucker et al. [222] observed that isolates of *Trichoderma* spp. were involved in effective protection of wood against certain basidiomycetes.

### 5.6. Agricultural and Horticultural Applications

Numerous *Trichoderma* spp. have also been used to protect fruits and vegetables of commercial significance throughout post-harvest storage. For example, Mortuza and Ilag [223] employed 10 isolates of *T. harzianum* and *T. viride* against *Lasiodiplodia theobromae* (fruit rot pathogen of banana). Similarly, Batta [224,225] applied the invert-emulsion formulation of *T. harzianum* Rifai in opposition to apple blue mold infection to prevent post-harvest decay of fruit. *Trichoderma* spp. are well-recognized fungal antagonists of crop/seed pathogens. Management of *Colletotrichum truncatum*, causing brown blotch of cowpea, has been done via the pre-treatment of seeds in *T. viride* spore suspension [226].

## 6. Conclusions and Future Perspectives

Biocontrol might be well-described as the practice of biological organisms or genetically altered genes or their products to lessen the consequences of unwanted organisms and to support organisms, which seems to be beneficial for human beings. As discussed in this review, *Trichoderma* spp. are correctly renowned for their capacity to generate a broad range of antibiotic substances that have the potential to parasitize a wide array of pathogenic fungi in the rhizosphere. In addition, *Trichoderma* spp. synthesize several metabolites which have a substantial influence on plant growth, along with stimulation of localized and systemic resistance and stress tolerance in plants. The recognition of *Trichoderma* elicitors and effectors by plant receptors initiates the signaling and regulation of host genetic apparatus, which serves as a basis for these symbionts to induce the defense metabolism in their host.

Further research dealing with the biochemical and physiological bases through which *Trichoderma* spp. act as biocontrol agent against several lethal fungi is necessary for a wide, in-depth knowledge of this multitalented biocontrol agent. Moreover, for the purpose of integrated disease management, the compatibility of *Trichoderma* with chemical fungicides should be evaluated. The popularity of *Trichoderma*-based formulations among farmers for ecofriendly management of diseases should be enhanced. The ecological influence of comprehensive applications of a fungal species as well as their secondary metabolites for biocontrol should be assessed to confirm a database for the secure and sustainable usage of *Trichoderma*. Consequently, *Trichoderma* genomes can also serve as an extremely useful source of candidate genes for producing transgenic plants exhibiting tolerance to both biotic and abiotic stresses. Lastly, by taking into consideration all the information provided in this review, the use of *Trichoderma* species should be promoted as a valid alternative to pesticides in the era of a green economy which aims at promoting human health and environmental safeguarding.
